# Supplementary material for: Resting-State Functional Connectivity following Phonological Component Analysis: The Combined Action of Phonology and Visual Orthographic Cues
Source: Brain Sci. 2021 Nov 2;11(11):1458. doi: 10.3390/brainsci11111458 (PMC8615968; doi:10.3390/brainsci11111458)
Supplement: Supplementary file 1 [file brainsci-11-01458-s001.zip › brainsci-1403348-supplementary.pdf]

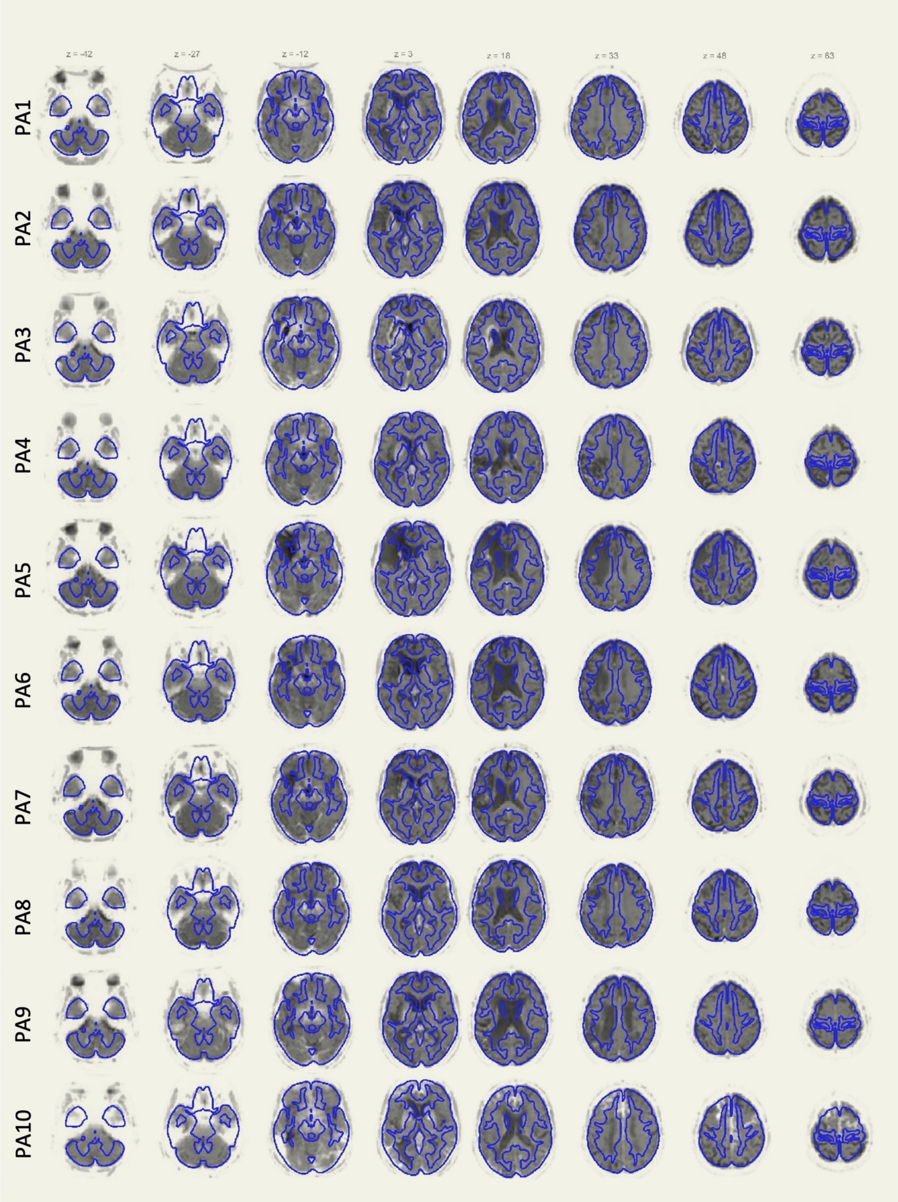

Figure S1. Registration quality comparison for each subject showing co-registered image overlapped to EPI template.
